# Evaluation of the Association between Single Nucleotide Polymorphisms of Metabolizing Enzymes with the Serum Concentration of Paracetamol and Its Metabolites

**DOI:** 10.3390/metabo12121235

**Published:** 2022-12-09

**Authors:** Kannan Sridharan, Ali Mohamed Qader, Mustafa Hammad, Anfal Jassim, Diab Eltayeb Diab, Betsy Abraham, Hasan M. S. N. Hasan, Sheikh Abdul Azeez Pasha, Shamik Shah

**Affiliations:** 1 Department of Pharmacology & Therapeutics, College of Medicine and Medical Sciences, Arabian Gulf University, Manama P.O. Box 26671, Bahrain; 2 Salmaniya Medical Complex, Manama P.O. Box 26671, Bahrain; 3 Department of Molecular Medicine, College of Medicine and Medical Sciences, Al-Jawhara Center, Arabian Gulf University, Manama P.O. Box 26671, Bahrain; 4 Intensive Care Unit, Salmaniya Medical Complex, Manama P.O. Box 26671, Bahrain; 5 Department of Nephrology, Salmaniya Medical Complex, Manama P.O. Box 26671, Bahrain

**Keywords:** paracetamol, acetaminophen, paracetamol sulphate, paracetamol glucuronide, pharmacogenetics

## Abstract

Intravenous paracetamol is a commonly administered analgesic and antipyretic in inpatient settings. Paracetamol is metabolized by cytochrome P450 (CYP) enzymes followed by conjugating enzymes to mainly glucuronide but to a lesser extent, sulphate metabolites, and oxidative metabolites. Single nucleotide polymorphisms (SNPs) in the CYP enzymes result in modified enzymatic activity. The present study was carried out to evaluate the prevalence of SNPs related to paracetamol metabolism and principal metabolites in critically ill patients, and those with chronic kidney disease. The present study is a cross-sectional study carried out in adults (>21 years) requiring intravenous paracetamol as part of their standard of care. Details regarding their demographics, and renal and liver function tests were collected. Blood was withdrawn for the analysis of paracetamol and their metabolites, and the SNPs of key CYP enzymes. Paracetamol/paracetamol glucuronide (P/PG), paracetamol/paracetamol sulphate (P/PS) and PG/PS were estimated. Acute liver injury (ALI) and renal dysfunction were defined using standard definitions. We observed a significant prevalence of SNPs in CYP1A2*1C, CYP3A4*3, CYP1A2*1K, CYP1A2*6, CYP2D6*10, and CYP2E1*2 amongst the 150 study participants. Those with CYP1A2*6 (CC genotype) were observed with significantly lower PG and PS concentrations, and a higher P/PS ratio; CYP2D6*10 (1/1 genotype) with a significantly lower PG concentration and a higher P/PG ratio; and CYP1A2*1K (CC genotype) was observed with a significantly higher PG/PS ratio. Good predictive accuracies were observed for determining the SNPs with the cut-off concentration of 0.29 μM for PS in determining CYP1A2*1K, 0.39 μM for PG and 0.32 μM for PS in determining CYP1A2*6 genotype, and 0.29 μM for PG in determining the CYP2D6*10 genotype. Patients with renal dysfunction were observed with significantly greater concentrations of paracetamol, PG and P/PS, and PG/PS ratios, with a lower concentration of PS. No significant differences were observed in any of the metabolites or metabolite ratios in patients with ALI. We have elucidated the prevalence of key CYP enzymes involved in acetaminophen metabolism in our population. Alterations in the metabolite concentrations and metabolic ratios were observed with SNPs, and in patients with renal dysfunction. Population toxicokinetic studies elucidating the dose-response relationship are essential to understand the optimized dose in this sub-population.

## 1. Introduction

Paracetamol is the commonly administered analgesic and antipyretic accounting for use in around 65% of critically ill patients [1]. Paracetamol use has been associated with a significant reduction in mortality (adjusted odds ratio: 0.6, 95% confidence interval (CI): 0.53 to 0.68) [1]. The analgesic and antipyretic actions of paracetamol are primarily attributed to its inhibition of the cyclooxygenase enzyme by binding on its peroxidase site [2]. Recent studies also indicate the activation of transient receptor potential vanilloid 1 and cannabinoid 1 receptors for its therapeutic effect [3]. The general thought that paracetamol is safe has been challenged from recent evidence relating it to a high risk of cardiovascular adverse events, gastrointestinal adverse events, renal impairment, and death [4].

Cytochrome P 450 (CYP) enzymes in the liver are primarily responsible for the phase 1 metabolism of paracetamol. Paracetamol is then conjugated to sulphate and glucuronide metabolites with a trivial fraction to oxidation product, chiefly by CYP2E1, CYP1A2, CYP3A4, and CYP2D6 enzymes [5]. Most of the metabolites are transported to kidneys through the blood stream from the liver for urinary excretion [6]. Hydroxylation and deacetylation are the other metabolizing pathways for paracetamol [7]. Previously, we evaluated the association of key CYP enzymes in the neonatal population and urinary sulphate and glucuronide metabolites, and observed the prevalence of single nucleotide polymorphisms (SNPs) as follows: heterozygous CYP1A2*1C (32.8%), heterozygous CYP1A2*1K (3.3%), heterozygous (27.8%) and homozygous (3.7%) CYP2D6*10, heterozygous CYP2E1*5B (3.7%), heterozygous (12.3%) and homozygous (3.3%) CYP3A4*1B, and CYP3A5*7 (5.5%) [8]. SNPs in CYP2D6 and CYP1A2 were recently attributed with hepatic toxicity [9]. Critically ill patients are at high risk of drug-induced-livery injury [10]. Additionally, in cases of paracetamol overdose, the conjugation pathways get saturated, and paracetamol is subsequently metabolized to N-acetyl-p-benzoquinone imine, a potential hepatotoxic metabolite [11]. There is a dearth of literature evaluating the association between the relevant SNPs with serum concentrations of paracetamol and its metabolites following intravenous administration. Hence, we carried out the present study to evaluate the prevalence of SNPs related to paracetamol metabolism, and principal metabolites in patients admitted who were critically ill, and patients with chronic kidney disease.

## 2. Experimental Design

### 2.1. Study Design and Ethics

This cross-sectional study was carried out between December 2021 and July 2022 in the Intensive Care Unit and Department of Nephrology, Salmaniya Medical Complex, Manama, Kingdom of Bahrain. Approvals from the Ethics Committee of College of Medicine and Medical Sciences, Arabian Gulf University (number: E032-PI-9/21) and Salmaniya Medical Complex (number: 126081121) were obtained. Written consent was obtained from study participants. The study was carried out in compliance to the latest Declaration of Helsinki guidelines.

### 2.2. Study Procedure

Adults (>21 years) requiring intravenous paracetamol (one gram) as part of a standard of care were recruited. The following details were obtained: age, sex, weight, diagnoses, drug-related details (name, dose, frequency, and route), laboratory test results (renal and liver function tests). One blood sample was collected from the intravenous canula after six hours (just before the second dose) following intravenous paracetamol administration, for the estimation of serum paracetamol, paracetamol glucuronide, and paracetamol sulphate concentrations. Additionally, one other blood sample was collected from each participant for the evaluation of SNPs. Acute liver injury (ALI) was diagnosed if alanine aminotransferase was elevated by at least 50% compared to admission values as per the recommendations from the Scottish and Newcastle antiemetic pretreatment for paracetamol poisoning study (SNAP) [12]. Similarly, we followed Kidney Disease: Improving Global Outcomes (KDIGO) guidelines, based on serum creatinine for defining those with renal dysfunction [13].

### 2.3. Method for Estimating Concentrations of Serum Paracetamol and Its Metabolites

The blood samples were stored in −80 °C pending analysis. We used high-performance liquid chromatography (HPLC) for measuring serum paracetamol, paracetamol glucuronide, and paracetamol sulphate concentrations with a slight modification of the method reported previously [14]. The HPLC system is from Waters^®^ (Milford, MA, USA), consist of e2695 pump, auto sampler and Waters^®^ UV/visible detector 2489, detector set at a wavelength of 254 nm. An isocratic mobile phase consisting of methanol and water 3:1 ratio, mobile phase circulated through Waters symmetry C18 5 µm 4.6 × 150 mm analytical column at a flow rate of 1.2 mL/min. The limit of detection for paracetamol metabolites was 0.1 μM and the linearity ranged between 0.1 and 10 μM. The limit of detection for serum paracetamol was 16 μM and the linearity ranged between 16 μM to 1324 μM. Percent coefficient of variation was 4.3, 4.5, and 4.8 for serum paracetamol glucuronide, paracetamol sulphate, and paracetamol, respectively.

### 2.4. Genotyping of Genetic Polymorphisms

QIAmp^®^ DNA Blood Mini Kit from Qiagen was used for extracting the genomic DNA from the peripheral blood leukocytes. We used Nanodrop spectrophotometer for measuring the DNA concentration. StepOne Plus^®^ real-time PCR system (Applied Biosystems; Foster City, CA, USA) was used for genotyping SNPs using the allelic discrimination method with the following commercially available TaqMan^®^ assays: rs2069514 (CYP1A2*1C), rs3813867 (CYP2E1*5B), rs4986910 (CYP3A4*3), rs72559170 (CYP2E1*2), rs56276455 (CYP1A2*3), rs12720461 (CYP1A2*1K), rs28399424 (CYP1A2*6), rs55785340 (CYP3A4*2), rs41303343 (CYP3A5*7), rs72552791 (CYP3A5*11), rs1065852 (CYP2D6*10), and rs2740574 (CYP3A4*1B).

### 2.5. Statistical Analysis

Descriptive statistics were used for mentioning the demographic variables. P/PG ratio was calculated by dividing the paracetamol concentrations over paracetamol glucuronide; P/PS ratio by dividing the paracetamol concentration upon paracetamol sulphate; and PG/PS by dividing paracetamol glucuronide upon paracetamol sulphate. Chi-square test of association was used for analyzing categorical variables. Numerical variables were evaluated for their distribution using Kolmogorov-Smirnov test and were analyzed using Mann-Whitney U test or Kruskal-Wallis test. Receiver operating characteristics (ROC) curves were plotted for the serum concentrations of paracetamol metabolites and the metabolite ratios for SNPs that were significantly associated. The diagnostic accuracies were categorized based on area under the curve (AUC) as follows: 0.9 to 1—excellent; 0.8 to 0.9—very good; 0.7 to 0.8—good; 0.6 to 0.7—sufficient; 0.5 to 0.6—bad; <0.5—not useful. Cut-off values of the metabolites that were significantly associated were estimated using Youden’s index. A *p*-value of <0.05 was considered significant. The SPSS version 26 (IBM Corp. Released 2019. IBM SPSS Statistics for Windows, Version 26.0. Armonk, NY, USA: IBM Corp.) was used for statistical analysis. As there was no data regarding the association between the SNPs evaluated in the present study and the serum concentrations of paracetamol and its metabolites, we did not estimate the sample size.

## 3. Results

### 3.1. Demographic Characteristics

One hundred and fifty participants were recruited, and a summary of their demographic characteristics is listed in Table 1. The majority of the study participants were middle-aged adults, with a near balance in the gender profile, with normal hepatic and renal functions. The following were the diagnoses observed: systemic hypertension (*n* = 85), diabetes mellitus (*n* = 75), end stage renal disease (*n* = 50), cerebrovascular accident (*n* = 19), road traffic accident (*n* = 11), post-surgical complications (*n* = 15), sickle cell disease with vaso-occlusive crisis (*n* = 10), three each with seizure disorder, Guillan-Barre syndrome and sepsis, two each with acute coronary syndrome and lupus nephritis, and one each with non-Hodgkin’s lymphoma, Hodgkin’s lymphoma, immune thrombocytopenic purpura, thalassemia, pulmonary embolism, renal vein thrombosis, burn with inhalational lung injury, pneumonitis, colon adenocarcinoma, intestinal bowel obstruction, ruptured ectopic pregnancy, diabetic ketoacidosis, diabetic foot, brain abscess, and pyelonephritis.

### 3.2. Genetic Polymorphisms

**Figure 1:**
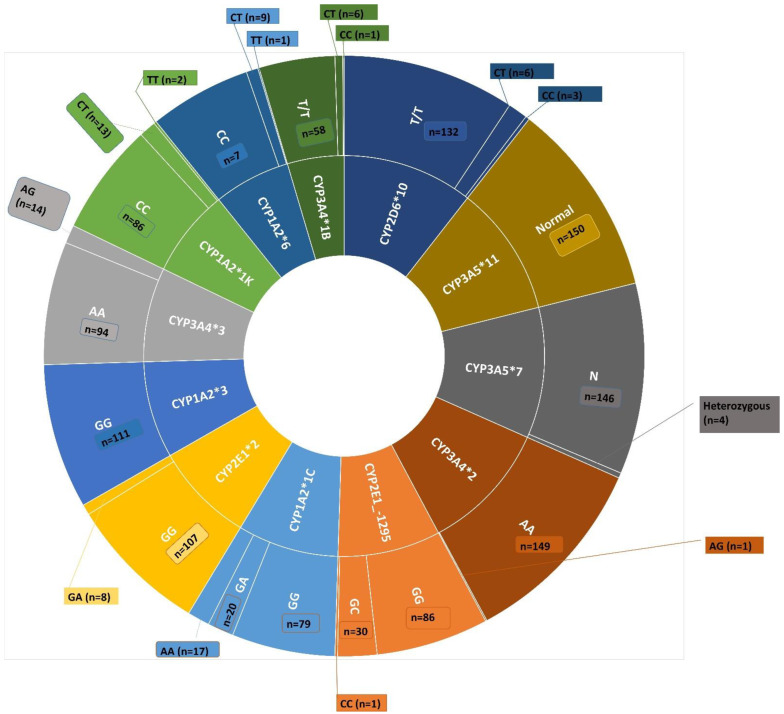
Prevalence of SNPs amongst the study participants. The inner circles represent the CYP enzymes evaluated in this study. The specific SNPs for each of the CYP enzymes are mentioned in the outer circles including the number of patients observed with each polymorphism.

The prevalence of SNPs amongst the study participants is depicted in Figure 1. No significant differences were observed in the frequency of paracetamol administration between genotypes, except for CYP3A4*2 where individuals with AA (*n* = 1) were observed with thrice daily compared to those with AG (once daily—33; twice daily—13; thrice daily—15; and four times daily—88) and was significantly different (*p* = 0.04).

**Figure 2:**
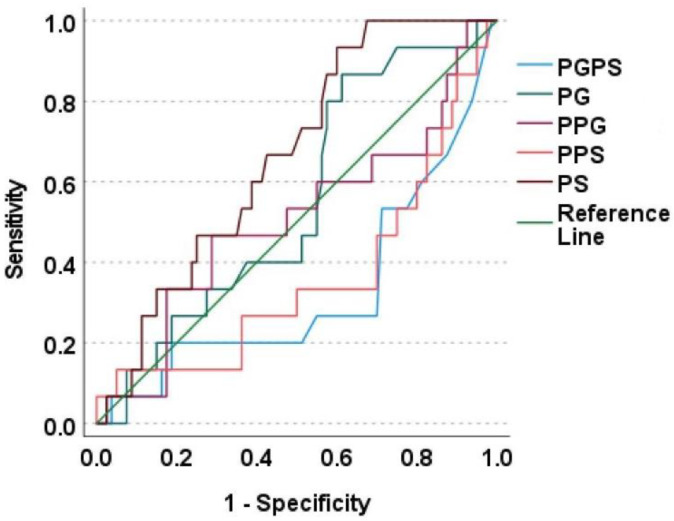
ROC plot for paracetamol metabolites with CYP1A2*1K. This is a ROC plot where the diagonal green line indicates the reference line. The brown line above the reference line represents the predictive ability of paracetamol sulphate. All other metabolites were observed with their lines falling below the reference line thereby they do not significantly predict the SNP. PGPS—Paracetamol glucuronide/Paracetamol sulphate ratio; PG—Paracetamol glucuronide; PPG—Paracetamol/Paracetamol glucuronide ratio; PPS—Paracetamol/Paracetamol sulphate ratio; PS—Paracetamol sulphate.

**Figure 3:**
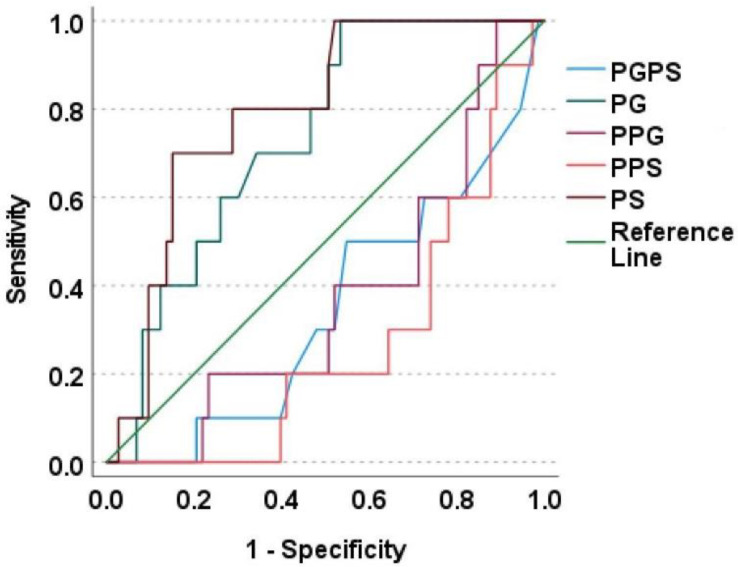
ROC plot for paracetamol metabolites with CYP1A2*6. This is a ROC plot where the diagonal green line indicates the reference line. The brown line above the reference line represents the predictive ability of paracetamol sulphate as like the dark green line that indicates paracetamol glucuronide metabolite. All other metabolite ratios were observed with their lines falling below the reference line thereby they do not significantly predict the SNP. PGPS—Paracetamol glucuronide/Paracetamol sulphate ratio; PG—Paracetamol glucuronide; PPG—Paracetamol/Paracetamol glucuronide ratio; PPS—Paracetamol/Paracetamol sulphate ratio; PS—Paracetamol sulphate.

**Figure 4:**
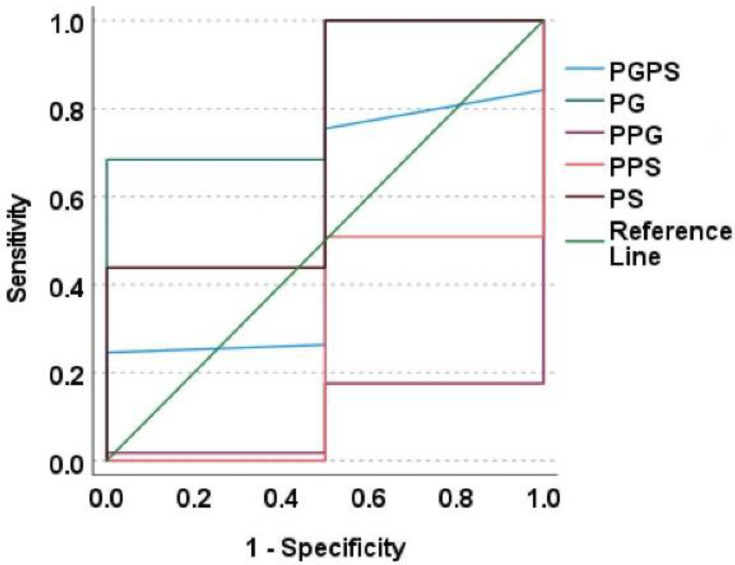
ROC plot for paracetamol metabolites with CYP2D6*10. This is a ROC curve where the diagonal green line indicates the reference line. The dark green line above the reference line represents the predictive ability of paracetamol glucuronide. All other metabolites/metabolite ratios were observed with their lines falling below the reference line thereby they do not significantly predict the SNP. PGPS—Paracetamol glucuronide/Paracetamol sulphate ratio; PG—Paracetamol glucuronide; PPG—Paracetamol/Paracetamol glucuronide ratio; PPS—Paracetamol/Paracetamol sulphate ratio; PS—Paracetamol sulphate.

The associations between SNPs and serum concentrations of paracetamol, paracetamol glucuronide and sulphate, and their ratios, are mentioned in Table 2. Those with CYP1A2*6 (CC genotype) was observed with significantly lower PG, PS concentrations and higher P/PS ratio, CYP2D6*10 (1/1 genotype) with significantly lower PG concentrations and higher P/PG ratio, and CYP1A2*1K (CC genotype) was observed with a significantly greater PG/PS ratio. ROC plots for the metabolites and metabolite ratios with the above-mentioned SNPs are depicted in Figure 2, Figure 3 and Figure 4. PG concentrations were significantly predicting CYP1A2*6 (good accuracy) and CYP2D6*10 (good accuracy) while PS significantly predicted CYP1A2*1K (good accuracy) and CYP1A2*6 (good accuracy) (Table 3). The cut-off concentrations for PS in determining CYP1A2*1K genotype was 0.29 μM, 0.39 μM for PG and 0.32 μM for PS in determining CYP1A2*6 genotype, and 0.29 μM for PG in determining CYP2D6*10 genotype.

### 3.3. Paracetamol and Paracetamol Metabolite Concentrations

**Figure 5:**
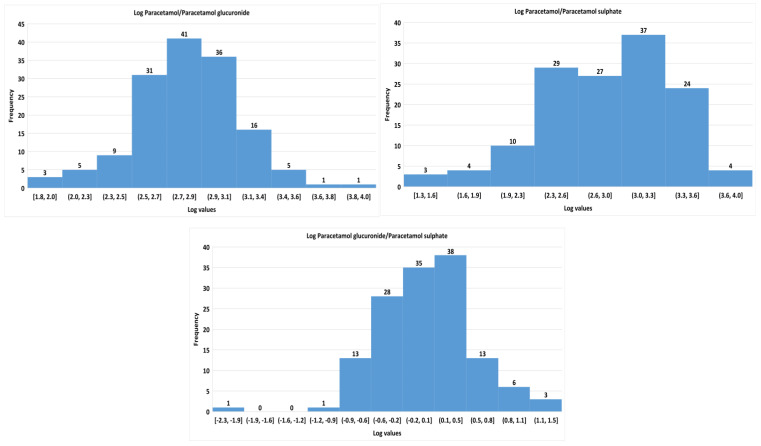
Frequency histogram of the metabolite ratios in the study population. These figures are the frequency histograms of log paracetamol upon paracetamol glucuronide (**top left**), log paracetamol upon paracetamol sulphate (**top right**), and log paracetamol glucuronide upon paracetamol sulphate (**bottom**). The vertical bars indicate the number of individuals observed with the specified log values of the ratios. The distribution looks similar for both paracetamol upon their metabolites while a bimodal distribution was observed for paracetamol glucuronide upon paracetamol sulphate.

In the study population, the median (range) concentrations of serum paracetamol were 314.2 (0–2745.2) μM, paracetamol glucuronide was 0.5 (0.1–10.8) μM, paracetamol sulphate was 0.4 (0.1–31.3) μM, P/PG ratio was 697.2 (64.8–6201.6), P/PS ratio was 831.8 (18.5–6950), and PG/PS ratio was 1.2 (0.006–20.3). The frequency histograms of the log paracetamol upon metabolites and between PG and PS are depicted in Figure 5. Bimodal distribution was observed in the log PG/PS ratio while other ratios showed unimodal distribution. The one participant with −2.3 log PG/PS ratio was observed to have CYP2D6*10 SNP.

Fifty patients had renal dysfunction. Patients with renal dysfunction were observed with significantly higher concentrations of paracetamol and PG but lower PS (Table 4). Consequently, P/PS and PG/PS ratios were significantly higher in patients with renal dysfunction (Table 4). Sixteen participants were observed with ALI. However, no significant differences were observed between those with ALI and those without (Table 4).

## 4. Discussion

### 4.1. Key Findings

The present study was carried out to explore the association between key SNPs of metabolizing enzymes with serum paracetamol, paracetamol metabolites, and metabolite ratios in 150 patients. We observed a significant prevalence of SNPs in CYP1A2*1C, CYP3A4*3, CYP1A2*1K, CYP1A2*6, CYP2D6*10, and CYP2E1*2. Those with CYP1A2*6 (CC genotype) was observed with a significantly lower PG and PS concentrations, and a higher P/PS ratio, CYP2D6*10 (1/1 genotype) with significantly a lower PG concentration and a higher P/PG ratio, and CYP1A2*1K (CC genotype) was observed with a significantly higher PG/PS ratio. Good predictive accuracies were observed for determining the SNPs with the cut-off concentration of 0.29 μM for PS in determining CYP1A2*1K, 0.39 μM for PG and 0.32 μM for PS in determining CYP1A2*6 genotype, and 0.29 μM for PG in determining CYP2D6*10 genotype. Patients with renal dysfunction were observed with significantly greater concentrations of paracetamol, PG and P/PS and PG/PS ratios, with a lower concentration of PS. No significant differences were observed in any of the metabolites or metabolite ratios in patients with ALI.

### 4.2. Comparison with the Existing Literature

The prevalence of SNPs identified in the present study in the Arab population are similar to previous reports [15,16,17,18]. CYP3A4, CYP2D6, and CYP1A2 enzymes have been implicated in the formation of reactive metabolites that lead to hepatotoxicity [19]. Paracetamol-induced hepatotoxicity mainly results from the oxidative metabolite that is mainly synthesized by CYP3A4 followed by CYP2E1, CYP1A2, and CYP2D6, as evidenced from in vitro studies [20]. In the present study, we did not observe any significant influence by CYP3A4, but from other enzymes. However, several other studies indicate CYP2E1 to be the predominant predictor of hepatotoxicity due to paracetamol [21]. CYP2E1 had a lower Km (higher affinity) compared to CYP1A2 enzyme [22]. Although higher metabolites were observed in patients with ALI in a previous study [23], we did not observe any significant difference in the present study that is possibly due to a fewer number of patients with ALI in the present study. Although SNPs are observed in sulphotransferases and glucuronyl transferases, they are not well elucidated and their influence on paracetamol metabolism is not yet known [24].

We observed significantly higher concentrations of paracetamol and glucuronide conjugate in patients with renal dysfunction. Renal dysfunction has been observed to result in reduced hepatic clearance by an extent of around 30% [25]. Sulfation pathway has been shown to be saturable at therapeutic doses and so sulphate conjugates have been debated to play a limited role in hepatotoxicity [26]. Patients with renal dysfunction have been shown to have reduced tubular sulphate reabsorption and fractional sulphate reabsorption compared to healthy humans [27]. Hence, endogenous sulphur pool is likely to be reduced in this population that possibly could explain the reduced quantity of PS metabolites. However, a few other studies have also observed higher PS conjugates in patients with renal dysfunction. Martin et al. observed significantly higher mean daily plasma concentrations of the sulphate and glucuronide conjugates of paracetamol in patients with chronic renal failure compared to healthy participants [28]. Prescott et al. observed significantly higher concentrations of paracetamol glucuronide and sulphate conjugates in patients with moderate renal failure and in dialysis patients [29]. In the latter group, the concentrations did not decline even after 24 h. Although paracetamol-induced hepatotoxicity mainly results from oxidative product (n-acetyl-p-benzoquinone imine), recently sulphate conjugate has also been observed to play a key role [30]. Additionally, we observed that serum paracetamol concentrations were significantly higher in patients with renal dysfunction that may result in the production of excess toxic oxidative metabolite. Hence, it is prudent to consider reducing dose and/or dosing frequency in this population.

This is the first study evaluating the influence of SNPs in CYP enzymes with serum concentrations of paracetamol, and its principal metabolites in critically ill and patients with renal dysfunction. However, the study is limited in including critically ill patients with a diverse disease profile and hepatic functions. Potentially, more insights on the impact of SNPs and metabolism of paracetamol can be attained in future studies limiting the study participants with narrow diagnosis and liver functions. Additionally, the impact on the pharmacodynamic effects namely, the antipyretic and analgesic effect shall also be evaluated in future studies.

## 5. Conclusions

We have elucidated the prevalence of key CYP enzymes involved in acetaminophen metabolism in our population. Alterations in the metabolite concentrations, and metabolic ratios were observed with SNPs and in patients with renal dysfunction. Population toxicokinetic studies elucidating the dose-response relationship are essential to understand the optimized dose in this sub-populations.

## Figures and Tables

**Table 1:** Demographic characteristics of study participants (*n* = 150).

Variables	Values
Age (years)	49 (21–91)
Male:Female (n)	82:68
Body weight (kg)	75 (43.6–166.7)
Serum total protein (g/L)	64 (31–95)
Serum albumin (g/L)	35 (18–53)
Serum bilirubin (mg/dl)	10 (2–265)
Serum alkaline phosphatase (IU/L)	98 (34–513)
Serum alanine transaminase (IU/L)	23 (8–2835)
Serum gamma-glutamyl transaminase (IU/L)	36 (6–650)
Serum creatinine (µmol/L)	113 (15–1857)

Values are mentioned in median (ranges).

**Table 2:** Association between the SNPs and serum concentrations of paracetamol and its metabolites.

SNPs		P (μM)	PG (μM)	PS (μM)	P/PG	P/PS	PG/PS
CYP2E1*5B	GG	320.9 (0–2745.2)	0.6 (0.1–6.8)	0.4 (0.1–31.3)	636.5 (64.8–6201.6)	909.2 (34.8–6949.9)	1.2 (0–19)
	GC	337.4 (86–740.9)	0.5 (0.1–1.9)	0.5 (0.1–5.2)	705 (306.5–3568.6)	524 (95–4931.2)	1.1 (0.2–4.8)
	CC	486.2 (205.1–767.3)	1.1 (0.3–1.9)	3 (0.6–5.3)	560.9 (399.7–722.1)	233 (144.5–321.4)	0.4
	*p*-values	0.8	0.5	0.3	0.8	0.1	0.1
CYP1A2*C	AA	357.2 (105.8–1587.6)	0.7 (0.2–1.9)	1.1 (0.1–6)	604.2 (107.3–1578.1)	416.7 (71.5–2898.7)	0.7 (0.2–4.8)
	AG	387 (125.7–740.9)	0.4 (0.2–1.4)	0.4 (0.2–5.2)	823.6 (306.5–1579.1)	956.6 (110.6–2205)	1 (0.2–4.6)
	GG	297.7 (0–2745.2)	0.6 (0.1–6.8)	0.4 (0.1–31.3)	612.5 (64.8–6201.6)	800 (34.8–5277.1)	1.3 (0–19)
	*p*-values	0.7	0.3	0.06	0.3	0.2	0.2
CYP3A4*3	AA	344 (0–2745.2)	0.6 (0.1–6.8)	0.4 (0.1–31.3)	636.5 (64.8–6201.6)	802.4 (34.8–6949.9)	1.2 (0–19)
	AG	393.6 (125.7–707.8)	0.5 (0.2–1.2)	0.6 (0.2–5.2)	662.9 (306.5–1579.1)	457.8 (71.5–2898.7)	0.7 (0.2–4.8)
	*p*-values	0.8	0.6	0.4	0.8	0.2	0.07
CYP2E1*2	GG	330.8 (0–2745.2)	0.6 (0.1–6.8)	0.4 (0.1–31.3)	637.5 (64.8–6201.6)	822.8 (34.8–6949.9)	1.2 (0–19)
	GA	423.4 (125.7–992.3)	0.4 (0.2–1.4)	0.6 (0.2–2.5)	836.3 (306.5–1579.1)	460 (203.4–2898.7)	0.7 (0.2–4.8)
	*p*-values	0.7	0.7	0.6	0.3	0.6	0.2
CYP1A2*3 (GG)		357.2 (0–2745.2)	0.6 (0.1–6.8)	0.4 (0.1–31.3)	695.6 (64.8–6201.6)	804.7 (34.8–6949.9)	1.2 (0–19)
CYP1A2*1K	CC	320.9 (0–2745.2)	0.6 (0.1–6.8)	0.4 (0.1–31.3)	668.2 (64.8–6201.6)	851.8 (34.8–5277.1)	1.3 (0–19)
	CT	403.5 (125.7–2745.2)	0.5 (0.2–2.4)	0.6 (0.2–3.1)	703.7 (306.5–2385.1)	510.6 (71.5–6949.9)	0.7 (0.2–10.4)
	TT	539.1 (310.9–767.3)	1.1 (0.3–1.9)	3.2 (1.1–5.3)	793.2 (399.7–1186.7)	216.9 (144.5–289.2)	0.3 (0.2–0.4)
	*p*-values	0.7	0.8	0.07	0.9	0.1	0.05 *
CYP1A2*6	CC	310.9 (112.5–1845.6)	0.5 (0.1–4.5)	0.4 (0.1–31.3)	722.1 (64.8–6201.6)	1016.6 (34.8–5277.1)	1.3 (0–19)
	CT	568.9 (178.6–992.3)	0.8 (0.4–1.9)	1.4 (0.3–2.7)	504.4 (323.6–1190.1)	514.9 (71.5–1378.1)	1.2 (0.2–2.5)
	TT	767.3	1.9	5.3	399.7	144.5	0.4
	*p*-values	0.06	0.04 *	0.008 *	0.3	0.05 *	0.2
CYP3A4*2	AA	310.9 (0–2745.2)	0.5 (0.1–10.8)	0.4 (0.1–31.3)	695.6 (64.8–6201.6)	840.8 (18.5–6949.9)	1.2 (0–20.3)
	AG	992.3	1.4	1.93	703.7	514.9	0.7
	*p*-values	0.2	0.2	0.2	0.9	0.7	0.6
CYP3A5*7	Wild	314.2 (0–2745.2)	0.5 (0.1–10.8)	0.4 (0.1–31.3)	697.2 (64.8–6201.6)	831.8 (18.5–6949.9)	1.2 (0–20.3)
	Heterozygous	383.7 (238.1–1772.8)	0.5 (0.2–3.1)	0.4 (0.1–2.2)	835.2 (566.4–1591.8)	1983.4 (370.4–5277.1)	1.7 (0.3–9)
	*p*-values	0.5	0.8	0.4	0.4	0.2	0.5
CYP3A5*11(Wild)		314.2 (0–2745.2)	0.5 (0.1–10.8)	0.4 (0.1–31.3)	697.2 (64.8–6201.6)	831.8 (18.5–6949.9)	1.2 (0–20.3)
CYP2D6*10	1/1	271.2 (238.1–317.5)	0.2 (0.1–0.3)	0.4 (0.1–0.8)	1273.5 (1114.1–3568.6)	2656.9 (382.6–4931.2)	0.9 (0.3–1.4)
	1/2	258 (119.1–1091.5)	0.3 (0.1–1.2)	0.5 (0.1–31.3)	816.4 (306.5–6201.6)	356 (34.8–3166.8)	0.6 (0–2.7)
	2/2	231.5 (0–2745.2)	0.6 (0.1–6.8)	0.7 (0.1–8.6)	484.5 (64.8–2367.1)	392.1 (18.5–4378.3)	0.7 (0.1–19)
	*p*-values	0.8	0.01 *	0.4	0.008 *	0.5	0.9
CYP3A4*1B	TT	244.8 (0–2745.2)	0.5 (0.1–6.8)	0.7 (0.1–31.3)	536.4 (64.8–6201.6)	387.4 (18.5–4931.2)	0.7 (0–19)
	CT	238.2 (145.5–648.3)	0.3 (0.2–0.8)	0.4 (0.1–2.5)	895.7 (323.6–1591.8)	677.6 (71.5–3166.8)	0.7 (0.2–2.1)
	CC	205.1	0.51	1.3	402.9	156.3	0.4
	*p*-values	0.9	0.5	0.4	0.4	0.4	0.8

P—Paracetamol; PG—Paracetamol glucuronide; PS—Paracetamol sulphate; *—Statistically significant.

**Table 3:** Diagnostic accuracies as indicated by AUCs (95% CI) of paracetamol metabolites and their ratios with SNPs.

Paracetamol Metabolites and Their Ratios	SNPs		
	CYP1A2*1K	CYP1A2*6	CYP2D6*10
PG	0.6 [0.4–0.7]	0.7 [0.6–0.9] *	0.8 [0.6–1] *
PS	0.7 [0.5–0.8] *	0.8 [0.7–0.9] *	0.7 [0.3–1]
P/PG	0.5 [0.3–0.7]	0.4 [0.2–0.5]	0.1 [0.1–0.2]
P/PS	0.4 [0.2–0.5]	0.3 [0.1–0.5]	0.3 [0.1–0.6]
PG/PS	0.3 [0.2–0.5]	0.3 [0.2–0.5]	0.5 [0.1–0.9]

P—Paracetamol; PG—Paracetamol glucuronide; PS—Paracetamol sulphate; *—Statistically significant.

**Table 4:** Comparison of serum paracetamol, paracetamol metabolites, and metabolite ratios between those with renal dysfunction and those without.

Paracetamol, Paracetamol Metabolites and Their Ratios	Renal Dysfunction (*n* = 50)	No Renal Dysfunction (*n* = 100)	*p*-Values
P	509.4 (112.5–2745.2)	264.6 (0–2745.2)	<0.001 *
PG	0.8 (0.1–4.5)	0.4 (0.1–10.8)	<0.001 *
PS	0.3 (0.1–4.6)	0.6 (0.1–31.3)	0.007 *
P/PG	625.2 (92.3–2385.1)	711.4 (64.8–6201.6)	0.7
P/PS	1481.9 (78.1–6949.9)	441.6 (18.5–4931.2)	<0.001 *
PG/PS	2 (0.2–20.3)	0.7 (0–19)	<0.001 *
**Paracetamol, paracetamol metabolites and their ratios**	**ALI (*n* = 16)**	**No ALI (*n* = 134)**	***p*-values**
P	363.8 (119.1–972.4)	314.2 (0–2745.2)	0.7
PG	0.5 (0.1–1.9)	0.5 (0.1–10.8)	0.9
PS	0.3 (0.1–3.2)	0.5 (0.1–31.3)	0.9
P/PG	617 (115.9–2467.8)	851.8 (38.5–3262.2)	0.7
P/PS	851.8 (38.5–3262.2)	813.8 (18.5–6949.9)	0.9
PG/PS	1.1 (0.2–4.6)	1.2 (0–20.3)	0.9

P—Paracetamol; PG—Paracetamol glucuronide; PS—Paracetamol sulphate; *—Statistically significant.

## Data Availability

The data is available with the corresponding author and will be shared upon a reasonable request. Data is not publicly available due to privacy.
